# Exploring Medium- and Long-Term Respiratory and Functional Sequelae in Young Adults Post-COVID-19

**DOI:** 10.3390/medicina61010086

**Published:** 2025-01-07

**Authors:** Büşra Ülker Ekşi, Eylül Pınar Kısa, Özge Ertan Harputlu, Begüm Kara Kaya, Zeynep Hoşbay, Buket Akıncı

**Affiliations:** 1 Department of Physiotherapy and Rehabilitation, Graduate Education Institute, Biruni University, 34015 Istanbul, Turkey; busra.eksi@galata.edu.tr; 2 Department of Therapy and Rehabilitation Physiotherapy Program, Vocational School, Istanbul Galata University, 34430 Istanbul, Turkey; 3 Department of Ergotherapy, Faculty of Health Sciences, İstanbul Medipol University, 34810 Istanbul, Turkey; eylul.kisa@medipol.edu.tr; 4 Department of Physiotherapy and Rehabilitation, Institute of Graduate Studies, Istanbul University-Cerrahpasa, 34320 Istanbul, Turkey; ozge.ertan@iuc.edu.tr; 5 Department of Physiotherapy and Rehabilitation (English), Faculty of Health Sciences, Biruni University, 34015 Istanbul, Turkey; bkara@biruni.edu.tr; 6 Biruni University Research Center (B@MER), Biruni University, 34015 Istanbul, Turkey; zhosbay@biruni.edu.tr; 7 Department of Physiotherapy and Rehabilitation, Faculty of Health Sciences, Biruni University, 34015 Istanbul, Turkey

**Keywords:** long COVID-19, post-COVID, respiratory function, physical function

## Abstract

*Background and Objectives:* Long COVID-19 syndrome may cause difficulties in functionality during daily life in young people. Our objective was to investigate the respiratory and functional sequelae in young adults with asymptomatic or mild COVID-19 compared with healthy peers 3–6 months and 6–12 months after COVID-19 infection. *Materials and Methods:* Participants aged 18–25 who had COVID-19 within the last 3–6 months (Post-COVID Group 1, n = 25) and 6–12 months (Post-COVID Group 2, n = 25) and age–gender-matched healthy controls (n = 25) were included in this study. Respiratory functions and muscle strength were measured. Physical function was assessed with 6 min walking test (6MWT) and an Incremental Shuttle Walk Test (ISWT). The 1 min sit-to-stand test (1-MSTST) and hand grip strength (HGS) were used to assess muscle performance. Fatigue and dyspnea severity were questioned. *Results:* The FVC%pred (*p* = 0.023) and MEP (*p* = 0.034) were higher, and 1-MSTST repetitions were lower in Post-COVID Group-1 compared to Post-COVID Group-2 (*p* = 0.029). The PEF%pred (*p* = 0.025), MEP (*p* = 0.001), and ISWT distance were lower in Post-COVID Group-2 compared to healthy controls. The number of 1-MSTST repetitions and 6MWT distance were lower in Post-COVID Group-1 (*p* = 0.003, *p* = 0.001) and Post-COVID Group-2 (*p* = 0.003, *p* = 0.017) than in healthy controls. Exercise-induced blood lactate change during the ISWT, HGS, fatigue, and dyspnea were not significantly different between post-COVID groups and healthy controls. *Conclusions:* Young adults who pass asymptomatic or mild SARS-CoV-2 infection exhibit a decline in FVC%pred, PEF%pred, lower extremity muscle performance, and physical function within 3–6 months. In addition, the deterioration in respiratory and physical functions becomes apparent within 6–12 months.

## 1. Introduction

With a high death and morbidity rate, COVID-19, which is caused by the Severe Acute Respiratory Syndrome Coronavirus 2 (SARS-CoV-2), has had a devastating effect on people all over the world. COVID-19 is a viral infection primarily targeting the respiratory system, accompanied by shortness of breath, cough, fever, and malaise [1]. Although most of the patients recovered from the COVID-19 infection, it was reported that more than 70% of the patients had involvement in different body systems, and the symptoms continued four months after the first symptoms appeared [2]. These medium- and long-term effects are jointly known as the post-COVID-19 state or long COVID-19 syndrome [3]. The most common persistent symptoms of post-COVID-19 are fatigue, dyspnea, muscle weakness, joint pain, chest pain, palpitations, and cough [4]. These symptoms usually consist of heterogeneous sequelae that affect multiple organ systems. The severity of the acute illness and the rate of admission to the intensive care unit affects the frequency of symptoms and systemic involvement. After six months of COVID-19 infection, the most prevalent symptoms were muscle weakness or fatigue, anxiety and depression, and sleep disturbances [5]. Physical and functional impairment was observed in the evaluation of 3–6 months after COVID-19 infection [6]. Most follow-up studies have focused on hospitalized severe cases. Therefore, knowledge of disease mechanisms in less severely affected individuals is limited. It was emphasized that it is necessary to investigate the impact of young individuals with mild-to-moderate disease without hospitalization [7]. Zhou et al. indicated that sleep difficulties, dyspnea, fatigue, anxiety, and depression were noted in over one-third of non-severe cases at approximately 1-year follow-up. To completely understand the health consequences of viral infection, it was reported that non-serious cases should also be followed up [8].

The well-being and healthy development of the younger demographic, a significant portion of the populace, are crucial for societal progress over the long haul. Greenhalgh et al. not only emphasized the importance of complete recuperation and the resolution of long COVID-19 symptoms but also highlighted the necessity of re-attaining economic productivity [9]. Assessing the impact of COVID-19 on young adults, irrespective of disease severity, along with the prompt identification of risks for current or forthcoming chronic conditions, holds substantial significance for secondary prevention strategies. Based on all these results, long COVID-19 syndrome may cause difficulties in functionality during daily life in young people. People’s ability to use their capacity depends on the symptoms, especially fatigue and exertional malaise. So, it was reported that the effects of these symptoms on activities of daily living, returning to school/work, and expected clinical courses should be investigated [10].

Studies on COVID-19 have generally focused more on high-risk and older populations in the literature. The long-term effects of COVID-19 in young adults, and whether these effects may be permanent, are an area that warrants further research. So, the aim of this study was to investigate the respiratory functions, respiratory muscle strength, physical function, lower extremity muscle performance, fatigue, and dyspnea of young adults compared with healthy age–gender-matched peers in medium- (3–6 months) and long-term (6–12 months) after COVID-19 infection.

## 2. Materials and Methods

### 2.1. Study Design

This study was conducted in the Biruni University Physiotherapy and Rehabilitation Division from May 2021 to October 2022. Ethical approval was obtained from the Clinical Research Ethical Committee (Approval number: 2015-KAEK-47-21-03) and conducted according to the Declaration of Helsinki. All subjects provided written informed consent. This study was registered in ClinicalTrials.gov (Registration number: NCT04864132).

Based on our pilot study [11], the G*Power (3.1.3 version) Sample Size Calculator was used to determine this study’s sample size. Considering the difference in six-minute walking distance between young adults who recovered from COVID-19 (574.60 ± 47.45 m) and healthy peers (616.14 ± 49.22 m), each group’s required patient number was determined as 23 with 80% power (Type I error probability = 0.05, effect size = 0.859, two tails). Twenty-five subjects for each group were recruited for this study with a 20% withdrawal rate estimation.

### 2.2. Participants

This study included fifty participants aged 18–25 years who had been diagnosed with COVID-19, confirmed through a positive SARS-CoV-2 real-time reverse transcriptase-polymerase chain reaction (RT-PCR) test on nasal swabs, with a minimum of 12 weeks having elapsed since their diagnosis. The post-COVID subjects were categorized as Post-COVID Group 1 (3 months–6 months) and Post-COVID Group 2 (6 months–12 months) based on the time after positive RT-PCR. This study incorporated a healthy control group consisting of twenty-five subjects matched by age and gender, all of whom had no prior diagnosis of COVID-19 and were not subjected to quarantine due to high-risk exposure. Exclusion criteria for this study included any pre-existing cardiac, pulmonary, or systemic conditions, orthopedic or neurological impairments, participation in a rehabilitation program within the previous three months, uncooperative behavior, fever exceeding 38 °C, resting blood pressure outside the range of 90/60 mmHg to 140/90 mmHg, or an oxygen (O_2_) saturation level of 95% or lower. Individuals presenting symptoms potentially related to undiagnosed COVID-19 at the time of recruitment were excluded from this study. Subjects suspected of having undiagnosed COVID-19 were directed to the hospital for further evaluation and were not included in the study cohort. To ensure adherence to these criteria, all potential participants were informed about the inclusion and exclusion requirements during the invitation and initial assessment phases. Consequently, none of the participants exhibited such symptoms during recruitment. These measures were implemented to minimize confounding effects and to ensure the integrity of the study results.

In this study, a health application provided by the Ministry of Health was utilized to monitor COVID-19 high-risk exposure, confirmed diagnoses, quarantine status, and test results. Through this application, and with the necessary permissions, data regarding prior COVID-19 exposure, quarantine history, and test results of individuals in the healthy control group were accessed. Individuals with a history of high-risk COVID-19 exposure were excluded from this study. This approach ensured accurate classification of participants and minimized the risk of including individuals with potential COVID-19 exposure, thereby enhancing the validity of this study.

### 2.3. Outcome Measures

This study documented demographic data of the participants, such as age, gender, weight, height, body mass index (BMI), and smoking habits. The International Physical Activity Questionnaire-Short Form was used to measure the participants’ levels of physical activity (IPAQ-SF). Seven items make up the IPAQ-SF, and they assess the duration, nature, and frequency (in days) of various activities. The total metabolic equivalent task (MET) can be used to calculate physical activity scores. Subjects’ physical activity levels were separated into three groups based on the total MET scores: low (0–600 MET-minutes/week), moderate (600–3000 MET-minutes/week), and high (3000 MET MET-minutes/week and above) [12].

#### 2.3.1. Respiratory Functions

The subjects’ respiratory functions and muscle strength were assessed through measurements. Spirometric evaluations adhered to the standards set by the American Thoracic Society/European Respiratory Society. With a spirometer (Micro Quark, COSMED Omnia, Italy), each patient’s forced expiratory volume in the first second (FEV1), forced vital capacity (FVC), FEV1/FVC ratio, and peak expiratory flow (PEF) were measured. These assessments were performed in compliance with the ERS’s spirometry guidelines, especially tailored for the COVID-19 pandemic period [13,14,15]. A minimum 30 s rest period was observed between forced expiratory maneuvers. At least three acceptable maneuvers were considered, with the best two values for FEV1 and FVC being within 5% of each other. To ensure acceptability, the patient exhaled fully for at least 6 s or until a plateau is reached, and attention was paid not coughing during the maneuver.

The mouth pressures were measured to determine the respiratory muscle strength of the subjects (RP Check MD Diagnostic, UK). Maximal inspiratory pressure (MIP) was assessed from the residual volume, and maximal expiratory pressure (MEP) was assessed using the total lung capacity.

#### 2.3.2. Physical Functions

##### Exercise Capacity

The assessment of exercise capacity involved conducting a six-minute walking test (6MWT) and an Incremental Shuttle Walk Test (ISWT). The 6MWT adhered to the guidelines established by the American Thoracic Society/European Respiratory Society [16]. The 6MWT was conducted in accordance with ATS/ERS guideline (2002). To eliminate the learning effect in the 6-min walk test 6MWT, participants underwent a familiarization session before the actual test. This allowed them to understand the protocol and adjust to the test conditions. Additionally, we followed standardized procedures with appropriate rest periods (minimum 20 min) between tests to prevent fatigue from influencing results. These measures ensured that the test outcomes reflected true functional capacity rather than practice-related improvements. Subsequently, the distance covered in six minutes (6MWD) was determined. Parameters such as blood pressure (Beurer GmbH, Uttenweiler, Germany), heart rate, oxygen saturation (MaProlinx GmbH, Düsseldorf, Germany), along with levels of dyspnea and leg fatigue (using the modified Borg Scale), were monitored and recorded pre- and post-test.

The physical function was evaluated using the ISWT as well. The ISWT comprises of 12 levels (1.020 m) where patients walk at their maximal performance at the symptom limit. The precautions listed above for the 6MWT to eliminate the learning effect were also considered and applied during the ISWT. The walking pace is gradually raised over each level. During the ISWT, walking speed was determined by an audio signal while the patients were walking around two cones placed 0.5 m indented on both sides on a 10 m length, keeping in time to an external audible signal. The subjects continue until they are too breathless or unable to keep up with the required pace. The number of completed shuttles was noted in meters.

The subjects’ oxygen saturation, heart rate, blood pressure, dyspnea, and leg fatigue were recorded before and after the test. To evaluate exercise-induced fatigue, the subjects’ blood lactate levels were determined. Measurements were taken from blood drawn from the middle finger of the right hand, utilizing a portable lactate analyzer (Accutrend^®^ Plus, Roche Diagnostics, Rotkreuz, Switzerland), both before and after the ISWT. The blood samples were processed within 60 s, with results noted in mmol/L-1. All utilized needles were disposed of in medical waste containers.

##### Lower Extremity Muscle Performance

The “1 min sit-to-stand test (1-MSTST)” assessed performance of the lower limb muscles. Prior the test, participants were shown how to perform it correctly. Conducted using a 46 cm height chair without armrests, the participant was seated with knees and hips bent at 90 degrees, feet flat and spaced hip-width apart, and arms crossed at the shoulders. The integrity of each cycle from sitting to standing back to sitting was verified to ensure a full sit-to-stand-to-sit sequence was completed [17]. A stopwatch was used to track time, while two observers tallied and verified the repetition count.

##### Hand Grip Strength

Grip strength of the dominant hand was gauged using a Jamar Hand Dynamometer (Baseline). Participants were seated, with the test arm’s shoulder adducted and elbow bent at 90 degrees, while the forearm and wrist remained in a neutral alignment. The assessment involved two maximum isometric holds lasting 5 s each. The greatest recorded measurement was identified as the maximum grip strength, recorded as both peak strength (in kilograms) and grip strength (in kilograms per kilogram of body mass) [18].

#### 2.3.3. Fatigue Severity

Fatigue severity was questioned using the Fatigue Severity Scale (Intercorrelation coefficient (ICC) = 0.81, Cronbach’s α = 0.96). The Fatigue Severity Scale (FSS) was used to evaluate patients’ fatigue in daily functions. The scale consists of 9 questions. The total score is calculated with the arithmetic average of nine items. A high score indicates increased fatigue severity in daily functions [19].

#### 2.3.4. Dyspnea Severity

Dyspnea was evaluated using Dyspnea-12 Scale (ICC = 0.9, Cronbach’s α = 0.9) [20]. The Dyspnea-12 Scale measures the severity of dyspnea. Each of the 12 items on the scale has a rating between 0 and 3 [21].

The evaluations were conducted by researchers who were unaware of the group allocations. Sufficient rest intervals (minimum 20–30 min) were provided between the assessments to minimize the influence of fatigue on the test outcomes. During all tests and evaluations, there was a strict adherence to pandemic-appropriate materials, mask-wearing, social distancing, and hygiene protocols.

### 2.4. Statistical Analysis

The Statistical Package for the Social Sciences (SPSS) version 21.0 software program was used to conduct the statistical analysis. Descriptive statistics were presented as mean (standard deviation) or number (frequency). The Shapiro–Wilk test was used to test the normality of data distribution. A one-way analysis of variance (ANOVA) with Bonferroni correction was used to compare the groups for the continuous parameters. Dunnett’s T3 post hoc multiple comparison test was performed to detect the differences between the groups. The Wilcoxon signed-rank test was used to compare discrete variables, while the Chi-square test was used for categorical variables. A *p*-value < 0.05 was considered statistically significant.

## 3. Results

Seventy-five subjects were included in this study and completed all assessments. The assessments revealed no unfavorable occurrences.

The mean time after the positive RT-PCR test was 124.75 ± 27.52/days in Post-COVID Group 1 and 279.60 ± 70.93/days in Post-COVID Group 2. Eight subjects (32%) in Post-COVID Group 1 and seven (28%) in Post-COVID Group 2 did not receive any medical treatment during the acute infection. All subjects who had COVID-19 were treated at home. Fatigue (55% in Post-COVID Group 1, 70% in Post-COVID Group 2) and headache (45% in Post-COVID Group 1, 35% in Post-COVID Group 2) were the most common COVID-19 symptoms during the acute phase of the disease. Ground-glass opacities consistent with viral pneumonia were detected in radiological imaging in two (8%) subjects in Post-COVID Group 2.

Demographic features, vaccination, pulmonary functions, and respiratory muscle strength of the groups are shown in Table 1. The groups were similar regarding demographic characteristics, vaccination, and physical activity levels (*p* > 0.05). The FVC % pred was significantly higher in Post-COVID Group 1 compared to Post-COVID Group 2 (*p* = 0.023, Z = −2275). The PEF % pred (*p* = 0.025, 95% CI 1.23/23.59) and MEP (*p* = 0.001, Z= −3.316) were significantly lower in Post-COVID Group 2 compared to healthy controls. The MEP was also significantly lower in Post-COVID Group 2 compared to Post-COVID Group 1 (*p* = 0.034, Z= −2.125). The FEV1% pred, FEV1/FVC %, MIP % pred, and MEP % pred were all similar in the groups (*p* > 0.05 for all).

The comparisons of lower extremity muscle performance, fatigue, dyspnea, physical activity, and exercise capacity of the subjects are shown in Table 2. The number of 1-MSTST repetitions was significantly lower in Post-COVID Group 1 (*p* = 0.001, Z= −3.325) and Post-COVID Group 2 (*p* = 0.017, Z = −2388) than in healthy controls. In addition, 1-MSTST repetitions were significantly lower in Post-COVID Group 1 compared to Post-COVID Group 2 (*p* = 0.029, Z = −2187). 6MWD was found to be significantly lower in Post-COVID Group 1 (*p* = 0.003, 95% CI 13.14/75.83) and Post-COVID Group 2 (*p* = 0.003, 95% CI 12.41/75.83) compared to healthy controls. The ISWT walking distance and estimated VO2 max were significantly decreased in Post-COVID Group 2 compared to healthy controls. HGS, FSS, and Dyspnea-12 were not different between groups (*p* > 0.05 for all).

The comparison of 6MWT and ISWT parameters between groups is shown in Table 2. No significant difference between the groups was observed in the blood lactate change in the ISWT (*p* = 0.580). The SpO_2_ was significantly decreased after the ISWT in Post-COVID Group 2 compared to healthy controls (*p* = 0.038). The changes in heart rate, blood pressure, leg fatigue, and dyspnea perception during the 6MWT and ISWT were similar in all groups (*p* > 0.05). Desaturation was not observed in any subjects in the groups. COVID-19 symptoms and medication history of subjects who recovered from COVID-19 are shown in Table 3.

## 4. Discussion

This study indicated that FVC %pred, PEF %pred, lower extremity muscle performance, and physical function might deteriorate in 3 to 6 months, and the worsening in respiratory functions and physical function can be seen after 6 to 12 months in young adults who had asymptomatic or mild SARS-CoV2. However, we found that exercise-induced blood lactate change, HGS, fatigue, dyspnea, and physical activity levels in healthy young people after COVID-19 did not significantly differ from their peers who had no diagnosis of COVID-19.

The pulmonary system is affected due to chronic inflammation, epithelial destruction, damage of capillary and bleeding, endothelial damage, hyaline membrane formation, and alveolar septal fibrous proliferation in the long term after COVID-19 [22]. Normal spirometry was observed after mild COVID-19 [23], while lower FVC and FEV1 with the preserved FEV1/FVC ratio suggested restrictive patterns observed in patients who suffered from moderate-to-severe term, and long-term consequences are decreased respiratory muscle strength and worsening of respiratory functions over time [22,24,25]. In this study, FVC %pred and PEF %pred were lower in the medium term than their healthy peers and worsened in the 6–12 months. Our study aligns with previous studies that reported decreased PEF %pred compared to healthy groups [12,26]. It has been stated that the PEF value is related to airway width, lung tissue compliance, and expiratory muscle strength [15]. Raza et al. reported decreased PEF after physical exertion and reported that the impairment is not related to airway restriction or obstruction but reduced lung volume [27]. Therefore, the decrease in FVC %pred and PEF %pred observed in our study may arise from the unconfirmed persistent radiological changes or restriction pathologies.

Previous studies reported a significant—with an extensive confidence interval range—decrease in MIP [26,28,29] and MEP [26,28] in the long term in young adults with mild COVID-19. Corral et al. reported a change of at least 18 cmH_2_O, and 22.1% of that % predicted MIP represented the minimally clinically important difference in post-COVID subjects [30]. We observed a decreasing trend in MIP and especially MEP in post-COVID groups. However, the difference for MIP was below the reported MCID, and the % predicted values for MIP and MEP were not significantly different compared to healthy controls. In addition, the respiratory muscle strength of our study groups was higher in healthy or post-COVID-19 groups than in previous studies conducted with younger populations [17,28,29]. Therefore, the long-term impairment in respiratory muscle strength is unclear in young adults with mild or asymptomatic COVID-19, and it should be further investigated.

Cortes et al. used sit-to-stand test to assess patient’s physical capacity and exertional desaturation and reported a decrease in 1-MSTST repetitions (mean number 20.9 ± 4.8) one month after discharge in patients who survived COVID-19 pneumonia [17]. Baricich et al. found that 1-MSTST repetitions of COVID-19 survivors were lower (mean number 19.7 ± 7.3) than expected at 3 to 6 months from hospital discharge [6]. The physical recovery trajectories of hospitalized COVID-19 patients after various care pathways were described by Berentschot et al. They reported that the percentage of normative values of 1-MSTST and HGS reached in 3 months and improved from 6 to 12 months in rehabilitation and with no care after hospital discharge [31]. From a different perspective, Ora et al. examined the performance of middle-aged military personnel infected in the first and second waves after COVID-19 and showed that the 1 min sit-to-stand repetitions but the similar HGS of those infected in the first wave declined more [32]. In our study, the number of 1-MSTST repetitions was lower in the 3–6 months of the patients who had COVID-19 compared to the healthy controls, and improvement was observed after six months of COVID-19. We observe no difference in HGS between post-COVID groups and healthy controls. HGS weakness was observed in mild or moderate to severe COVID-19 patients in the 3–6 months [33,34]. Ramirez et al. suggested that muscle strength was mediated by an appendicular lean mass index, which explained 52% of the association for HGS in long COVID-19 syndrome [34]. The authors explained that the decreased muscle strength is related to hypoxia, malnutrition, medication, and impaired myoblast replication. Our sample group consisted of young subjects with no history of intensive care unit stay or prolonged immobilization due to COVID-19. Therefore, we did not expect a significant reduction in muscle mass. Based on our findings, it may be argued that preserved muscle mass may prevent weakness in HGS in younger adults.

The deteriorated physical function in COVID-19 survivors is not correlated with the severity of COVID-19 [35,36]. The assessment of exercise capacity and physical function was suggested even in young and asymptomatic subjects who had COVID-19 for determining long-term results of COVID-19 [37,38]. Previous studies that assessed the physical function using the 6MWT reported that an increase can be noted in the first six months after the infection. However, the recovery was not completed in physical function 1–2 years after infection [39]. In this study, the 6MWDs were in the normal range in all groups [40], but there was a noticeable significant difference (~45 m) between post-COVID subjects and their age–gender-matched peers. As an unexpected result, no significant improvement in physical function was observed 6–12 months after infection, despite relatively higher physical activity participation. The ISWT is another field test that evaluates maximal effort and is suggested to evaluate the physical function in post-COVID-19 patients [41]. It was reported that shorter ISWT distance is associated with post-COVID dyspnea [42]. According to 6MWT results, we found substantial decreases in ISWT distance and estimated VO_2_ max in the young adults 6–12 months after COVID-19 compared to healthy controls despite similar dyspnea perceptions within the groups. According to Evers et al., declining exercise capacity was linked to lower O_2_ pulse and peak but not to ventilatory or pulmonary vascular limitation. It was also not correlated with the initial severity of COVID-19. Additionally, we believe that diminished lower limb muscle strength and poor microcirculation [35,36].

Post-COVID dyspnea and fatigue are persistent problems after 12 months of non-severe COVID-19 [8]. In our study, the post-COVID groups presented similar disease characteristics regarding dyspnea and fatigue prevalence during the initial diagnosis. Self-reported dyspnea or fatigue severity was not different from healthy controls in post-COVID groups. Rinaldo et al. noted that patients with mild to critical COVID-19 reported lower-than-expected levels of dyspnea and fatigue in three months [36]. Also, previous studies reported exercise-induced dyspnea and fatigue after the 6MWT in the early post-discharge period in individuals with moderate to severe COVID-19 [35,36,39]. In line with previous studies, none of the subjects were desaturated during the test, and exercise-induced leg fatigue or dyspnea in physical function assessments were similar in all groups in our study. Additionally, we measured exercise-induced blood lactate change as an objective fatigue marker in the ISWT. Although blood lactate changes were higher and the ISWT distance was 14 meters shorter in post-COVID Group 2 compared to Group 1, no significant differences were observed between the post-COVID groups and healthy controls. Our findings align with Rinaldo et al., who reported no difference in peak lactate levels during CPET between subjects with normal and reduced exercise capacity [36]. On the other hand, we think that our result could postulate that a shorter walking distance could indicate that these patients may experience greater peripheral fatigue in the muscles, particularly in the lower limbs, which could contribute to early termination of the ISWT due to muscle fatigue rather than respiratory distress.

This study has several limitations. First, the effects of COVID-19 were examined in groups at 3–6 months and beyond six months, but it was not possible to track the same individuals over time. Additionally, the pre-COVID-19 status of participants regarding the outcome measures was unknown, limiting the ability to assess changes relative to baseline. While VO_2_ reference equations from previous studies were utilized, a cardiopulmonary exercise test (CPET) was not performed to directly measure exercise capacity. Furthermore, long COVID-19 is influenced by various confounding factors, and these may not have been fully accounted for in the analysis. Another potential confounder is that some individuals, despite being infected, may not have sought any medical care, which could affect this study’s findings. Lastly, a larger sample size would have enhanced the generalizability and statistical power of the results.

## 5. Conclusions

In conclusion, our results demonstrate that respiratory functions, lower extremity muscle performance, and physical function may affect young adults with asymptomatic or mild COVID-19 over three months to one year. The novel contribution of this study to the literature is the long-term effects of COVID-19 may become more prominent after six months or longer even in young adults. Therefore, the respiratory and functional sequels of SARS-CoV2 should be followed up, and prevention strategies should be implemented to avoid future comorbidities in young adults with prominent roles in productivity, active brain force, and the workforce.

## Figures and Tables

**Table 1:** The comparison of demographic features, spirometry, and respiratory muscle strength of the subjects.

	Healthy Controls n = 25	Post-COVID Group 1 n = 25	Post-COVID Group 2 n = 25	*p*
Age (years)	21.9 ± 0.96	21.3 ± 1.86	20.85 ± 1.49	0.078
Gender				
Female	14 (56%)	16 (64%)	17 (68%)	1.000
BMI (kg/m^2^)	21.92 ± 2.25	23.84 ± 3.99	22.21 ± 2.79	0.177
Time after positive PCR (days)	-	124.75 ± 27.52	279.60 ± 70.93	**<0.0001**
Vaccination				0.457
Sinovac	1 (4%)	1 (4%)	3 (12%)	
BioNTech	2 (8%)	1 (4%)	-	
Smoking status				0.478
Smoker	17 (70%)	14 (56%)	15 (60%)	
Non-smoker	8 (30%)	8 (30%)	6 (25%)	
Ex-smoker	-	3 (14%)	3 (14%)	
Physical activity level (IPAQ-SF)				0.686
Low	4 (15%)	4 (15%)	1 (5%)	
Moderate	17 (70%)	16 (65%)	16 (65%)	
High	4 (15%)	5 (20%)	8 (30%)	
FEV1 *	3.71 ± 0.49	3.42 ± 0.80	3.08 ± 0.28	**0.004**
FEV1%pred	91.62 ± 8.03	88.52 ± 12.16	86.88 ± 9.12	0.322
FVC *	4.42 ± 0.61	4.25 ± 0.87	3.71 ± 0.53	**0.006**
FVC%pred	93.87 ± 8.99	95.70 ± 6.90	81.94 ± 27.53	**0.032**
FEV1/FVC% *	0.84 ± 0.05	0.80 ± 0.07	0.84 ± 0.10	0.157
PEF	7.31 ± 1.52	6.15 ± 1.66	5.20 ± 0.84	**<0.0001**
PEF%pred *	82 ± 14.84	73.06 ± 15.62	69.58 ± 12.31	**0.024**
MIP (cmH_2_O) *	103.37 ± 19.15	95.23 ± 18.39	90.66 ± 17.46	0.072
MIP%pred *	0.95 ± 0.18	0.90 ± 0.15	0.90 ± 0.15	0.846
MEP (cmH_2_O) *	119.06 ± 23.39	109 ± 21.23	95.27 ± 14.98	**0.002**
MEP%pred *	0.60 ± 0.13	0.58 ± 0.08	0.55 ± 0.10	0.882

Data presented as mean ± standard deviation or n (%), * One-way ANOVA. BMI: Body mass index, PCR: Positive chain reaction, pred: predicted, FEV1: Forced Expiratory Volume in 1 second, FVC: Forced vital capacity, PEF: Peak expiratory flow, MIP: Maximal inspiratory pressure, MEP: Maximal expiratory pressure.

**Table 2:** The comparison of lower extremity muscle performance, hang grip strength, fatigue, dyspnea, physical activity, and physical functions of the subjects.

	Healthy Controls n = 25	Post-COVID Group 1 n = 25	Post-COVID Group 2 n = 25	*p*
6MWD (m) *	615.37 ± 40.88	570.88 ± 45.97	571.61 ± 32.54	**0.001**
Δ SpO_2_ (%)	−0.37 ± 0.78	−0.41 ± 0.97	−0.38 ± 0.92	0.956
Δ Heart rate (beat/min) *	46.37 ± 16.07	37.94 ± 19.42	39.83 ± 20.03	0.329
Δ BORG dyspnea	0.68 ± 1.15	0.52 ± 1.34	0.72 ± 1.44	0.823
Δ BORG leg fatigue	2.25 ± 2.58	2.41 ± 2.95	2.22 ± 2.58	0.984
ISWT walking distance (m)	582.63 ± 127.058	544.03 ± 108.56	530.90 ± 124.96	**0.026**
Δ SpO_2_ (%)	0 ± 0.97	−0.29 ± 0.62	−0.72 ± 1.20	**0.038**
Δ Heart rate (beat/min) *	58.56 ± 23.86	49.05 ± 25.05	44.22 ± 24.14	0.176
Δ BORG dyspnea	1.06 ± 1.95	2.11 ± 2.44	0.77 ± 1.14	0.170
Δ BORG leg fatigue	3.62 ± 2.19	3.35 ± 2.53	3 ± 2.51	0.666
Δ Blood lactate change (mmol/dL) *	1.75 ± 1.69	1.08 ± 1.98	1.48 ± 2.32	0.580
VO_2_ max	18.75 ± 3.17	17.79 ± 2.71	17.46 ± 3.12	**0.026**
1 min sit-to-stand test	32.14 ± 4.98	26.01 ± 4.01	28.82 ± 4.90	**0.001**
Dominant hand grip strength	42.25 ± 21.55	44.65 ± 26.15	40.92 ± 16.83	0.966
FSS	28.31 ± 9.96	29.05 ± 10.32	31.35 ± 11.39	0.613
Dyspnea 12	3.50 ± 4.77	3.20 ± 4.03	3.23 ± 2.64	0.635
Physical dyspnea	2.93 ± 3.53	3.05 ± 3.95	2.94 ± 2.35	0.552
Emotional dyspnea	0.56 ± 1.77	0.15 ± 0.48	0.29 ± 0.70	0.297

Data presented as mean ± standard deviation or n (%), * One-way ANOVA. FSS: Fatigue Severity Scale, 6MWD: Six Minutes Walking Distance, ISWT: Incremental Shuttle Walk Test, Δ: posttest–pretest, VO_2_ max: Maximum oxygen uptake.

**Table 3:** COVID-19 symptoms and medication history of subjects recovered from COVID-19.

COVID-19 Symptoms	Post-COVID Group 1 n = 25	Post-COVID Group 2 n = 25	*p*
**Fatigue**	14 (55%)	17 (70%)	0.327
Headache	11 (45%)	9 (35%)	0.519
Cough	10 (40%)	7 (28%)	0.507
Dyspnea	9 (35%)	7 (28%)	0.736
Muscle and joint pain	10 (40%)	9 (35%)	0.744
Secretion	6 (25%)	4 (16%)	0.429
Loss of smell and taste	7 (30%)	4 (16%)	0.256
Gastrointestinal problems	5 (20%)	4 (16%)	0.677
Fewer	5 (20%)	4 (16%)	0.677
**Medical treatment during acute infection**			
None	8 (32%)	7 (28%)	0.758
Hidroksiklorokin	-	4 (16%)	
Azitromisin	5 (20%)	5 (20%)	
Favipiravir	12 (48%)	9 (36%)	

Data presented as n (%).

## Data Availability

Data are contained within the article.
